# Mentalizing Deficits Constrain Belief in a Personal God

**DOI:** 10.1371/journal.pone.0036880

**Published:** 2012-05-30

**Authors:** Ara Norenzayan, Will M. Gervais, Kali H. Trzesniewski

**Affiliations:** 1 Department of Psychology, University of British Columbia, Vancouver, Canada; 2 Department of Human Development and Family Studies, University of California, Davis, Davis, California, United States of America; Ecole Normale Supérieure, France

## Abstract

Religious believers intuitively conceptualize deities as intentional agents with mental states who anticipate and respond to human beliefs, desires and concerns. It follows that mentalizing deficits, associated with the autistic spectrum and also commonly found in men more than in women, may undermine this intuitive support and reduce belief in a personal God. Autistic adolescents expressed less belief in God than did matched neuro-typical controls (Study 1). In a Canadian student sample (Study 2), and two American national samples that controlled for demographic characteristics and other correlates of autism and religiosity (Study 3 and 4), the autism spectrum predicted reduced belief in God, and mentalizing mediated this relationship. Systemizing (Studies 2 and 3) and two personality dimensions related to religious belief, Conscientiousness and Agreeableness (Study 3), failed as mediators. Mentalizing also explained the robust and well-known, but theoretically debated, gender gap in religious belief wherein men show reduced religious belief (Studies 2–4).

## Introduction

Belief in God and other supernatural agents is culturally and historically widespread, and is a deeply affecting aspect of human life [1]. Yet relatively little is known about the cognitive foundations of these complex sociocultural beliefs. Believers intuitively treat gods as intentional agents with mental states who enter into social relationships with humans, using supernatural powers to assuage existential concerns, respond to human desires, and monitor their social behaviour [1]–[5]. Cognitive theories therefore converge on the hypothesis that supernatural agent beliefs are partly rooted in ordinary human social cognition. Specifically, the social-cognitive capacity to represent and reason about minds-termed mentalizing, theory of mind, or mind perception [6], [7] -also enables the mental representation of God and other supernatural agents [2], [7]. If mentalizing supports the mental representation of supernatural agents, then mentalizing deficits associated with the autistic spectrum and also commonly found in men more than in women [6], [8], [9] may undermine intuitive support for supernatural agent concepts and reduce belief in God [1], [10]–[13]. Here we examine the hypothesis-long predicted, though currently untested- that mentalizing deficits constrain belief in God.

In neuroimaging studies, thinking about [14] and praying to [15] God activates brain regions implicated in mentalizing; thus mentalizing might be a necessary component of belief in God, without being a sufficient cause. When adults form inferences about God's mind, they show the same mentalizing biases that are typically found when reasoning about other peoples' minds [16]–[18]. Developmentally, children's reasoning about God's mental states, and about other non-physical agents, tracks the cognitive development of mentalizing tendencies [19], [20]. Finally, mentalizing is deficient at higher levels of the autism spectrum [8], [9], [21], [22], and interestingly men are both more likely to score high on the autism spectrum [23] and more likely to be non-believers [24]–[26]. These lines of evidence suggest that mentally representing supernatural beings (and their mental states) requires mentalizing capacities. This in turn implies that mentalizing deficits would constrain intuitive support for belief in God. Recent unpublished findings by Caldwell-Harris, Murphy, Velazquez, and McNamara (2011) provide some indirect support to this line of reasoning. Adults who reported being diagnosed with autism spectrum disorder were more likely than a neuro-typical comparison group to self-identify as atheist and less likely to belong to an organized religion.

### The Present Research

We used an individual differences approach to test three interrelated cognitive hypotheses: 1) the autistic spectrum is inversely related to belief in God, 2) mentalizing mediates this relation, and 3) mentalizing mediates the long-known, but theoretically ambiguous, gender gap wherein men show reduced religious belief than women. Study 1 compared religious belief in a sample of adolescents with clinical diagnoses of autism with a neuro-typical sample matched on relevant socio-demographic characteristics. Studies 2–4 replicated and extended our findings to three distinct non-clinical samples that measured autism as a continuous variable rather than as a clinical diagnosis. These studies also enabled formal mediation analyses, in which we tested our hypotheses with multiple mediation bootstrapping based on 9999 resamples [27], an analytic technique for simultaneously testing multiple potential mediators (thus avoiding the inferential pitfalls of sequential analyses). In all analyses where gender was included, it was coded (female = 0, male = 1), and all other variables were standardized. Studies 2–4 also controlled for additional socio-demographic and psychological variables related to autism, mentalizing, and religiosity that addressed several alternative explanations, which we evaluate in the General Discussion.

### Ethics Statement

For Studies 2–4, approval was obtained from the Behavioral Research Ethics Board (BREB) at the University of British Columbia, and all participants provided written informed consent (for participants who completed the study via the internet, consent was provided by clicking a designated button online; this was approved by the UBC BREB). For Study 1, the parent was given a letter of information and signed a written consent form and the child was given an assent form to read and sign. Approval for the study was obtained from the University of Western Ontario Research Ethics Board.

## Results and Discussion

### Study 1

In a logistic regression model with autism diagnosis and IQ predicting belief in God, autistic participants were only 11% as likely as neuro-typical controls to strongly endorse God, OR = .11, 95% CI = .01, .96, *Wald* = 3.98, *p* = .046, and IQ was unrelated to belief, OR = 1.01, 95% CI = .96, 1.06, *Wald* = .22, *p* = .64.

This study allowed comparison of individuals with autism diagnoses with a matched control group, but the small sample size rendered statistical mediation testing unfeasible. As an alternative, we entered IQ and parental ratings of adolescent mentalizing tendencies (the two were uncorrelated *r* = .15, *p* = .50) as independent predictors of belief in God. In this logistic regression model, only mentalizing was a significant predictor: for each standard deviation decrease in mentalizing, participants were only 21% as likely to strongly endorse God, OR = .21, 95% CI = .06, .73, *Wald* = 6.07, *p* = .01, and again IQ was not a significant predictor, OR = 1.03, 95% CI = .98, 1.08, *Wald* = .96, *p* = .33.

### Study 2

Study 2 utilized a Canadian student sample and measured belief in God, a standard self-report measure of the autism spectrum, and both mentalizing (The Empathy Quotient) and systemizing [28] as potential mediators. Empathy has been used extensively to detect individual differences in adult mentalizing tendencies, including perspective taking, interest in others' beliefs and desires, and understanding emotions. It is inversely correlated with autism and with being male [8], [28]. Systemizing assesses individual differences in abilities and interests concerning physical and rule-based systems. Because systemizing is positively correlated with autism and with being male [28], but is typically orthogonal to mentalizing, it was included as a second potential mediator. Neither mentalizing nor systemizing has religious content or share conceptual resemblance with the belief in God measure. We tested our two primary hypotheses with autism and gender (respectively) predicting belief in God, and mentalizing (Empathy) and systemizing as potential mediators.

**Figure 1:**
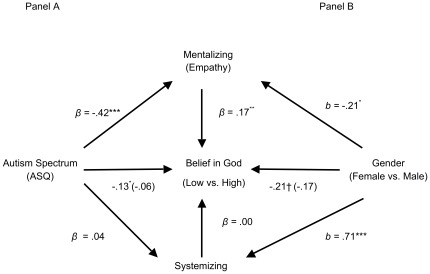
Study 2: Mentalizing, but not systemizing, mediated the effects of both autism spectrum (A) and gender (B) on belief in God (*N* = 327). †*p*<.10, *p<.05, **p<.01, ***p<.001. Note. OR = odds ratio; *β* = standardized beta; b = unstandardized beta. Values in parentheses are mediated effects. Autism Analysis Covariate: Gender. Gender Analysis Covariate: Autism Spectrum.

First, we tested our autism-related hypothesis (Fig. 1A). As hypothesized, higher autism scores predicted lower belief in God, *β* = −.13, *p* = .02 (controlling for gender). Critically, as predicted, mentalizing significantly mediated this relationship, b_MentalizingIndirect_ = −.07, 95% CI = −.14, −.01. Systemizing was not a significant mediator, b_SystemizingIndirect_ = .00, 95% CI = −.007, .009.

We next examined the effect of gender (Fig. 1B). Consistent with past research, there was a trend for men to report weaker belief in God than women (controlling for the autism spectrum), *b* = −.21, *p* = .08 (this gender gap was significant at *p* = .05 when autism was not controlled). As hypothesized, there was a significant indirect effect, such that men were lower in mentalizing, and in turn lower mentalizing predicted lower belief in God, b_MentalizingIndirect_ = .04, 95% CI = .003, .103; there was no significant indirect effect for systemizing, b_SystemizingIndirect_ = −.001, 95% CI = −.086, .084, even though gender more strongly predicted systemizing (*b* = .71) than mentalizing (*b* = −.21).

### Study 3

Study 3 replicated and extended findings from Study 2 to a broad national sample of American adults, controlled for a range of covariates of the autism spectrum and religious belief, and used an alternative measure of belief in and a personal relationship with God. We again tested mentalizing (The Empathy Quotient) and systemizing as mediators. In addition, we measured and tested two additional potential mediators. Two of the five basic facets of personality [29], Conscientiousness and Agreeableness, are reliably related to religious belief and involvement in both previous research [30], [31] and also in the current sample. Therefore it is plausible that they could also explain links between autism or gender and belief in God.

**Figure 2:**
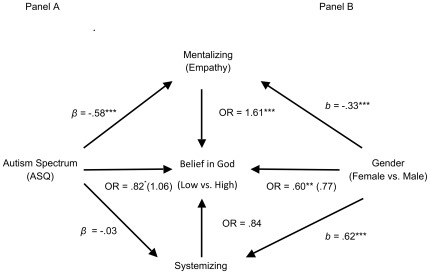
Study 3: Mentalizing, but not systemizing, mediated the effects of both autism spectrum (A) and gender (B) on belief in a personal God (*N* = 706). *p<.05, **p<.01, ***p<.001. Note. OR = odds ratio; *β* = standardized beta; b = unstandardized beta. Agreeableness, or Conscientiousness (not shown) also failed as mediators. Values in parentheses are mediated effects. Autism Analysis Covariates: Gender, Age, Education, Income, Religious attendance. Gender Analysis Covariates: Autism Spectrum, Age, Education, Income, Religious attendance.

In a logistic regression model with autism spectrum predicting belief in a personal God, controlling for gender, age, educational attainment, income, and frequency of religious attendance, for each standard deviation increase in autism scores, participants were only 80% as likely to strongly endorse a personal God, OR = .80, 95% CI = .66, .97, *Wald* = 5.25, *p* = .02. Mentalizing emerged again as a significant mediator of this relationship, b_MentalizingIndirect_ = −.20, 95% CI = −.37, −.04, while systemizing did not, b_SystemizingIndirect_ = −.007, 95% CI = −.03, .008 (Fig. 2A). Furthermore, neither of the two personality measures were significant mediators, b_ConscientiousnessIndirect_ = −.02, 95% CI = −.07, .02, b_AgreeablenessIndirect_ = −.10, 95% CI = −.20, .004.

In a separate logistic regression model, men were only 60% as likely to report strong belief in a personal God as women, controlling for the autism spectrum, age, income, educational attainment, and frequency of religious attendance, OR = .60, 95% CI = .41, .89, *Wald* = 6.55, *p* = .01. Replicating the pattern from Study 2, there was a significant indirect effect such that men were lower in mentalizing, and in turn lower mentalizing scores predicted lower belief in a personal God, b_MentalizingIndirect_ = −.15, 95% CI = −.28, −.06 (Fig. 2B); there was no significant indirect effect for any other potential mediator, b_SystemizingIndirect_ = −.007, 95% CI = −.03, .008, b_ConscientiousnessIndirect_ = −.02, 95% CI = −.07, .02, b_AgreeablenessIndirect_ = −.10, 95% CI = −.20, .004. It was noteworthy that systemizing again failed as a mediator of the gender effect, despite the fact that, once again, gender more strongly predicted systemizing (*b* = .62) than mentalizing (*b* = −.33) (Fig. 2B).

Finally, in a comprehensive logistic regression model predicting high belief in a personal God, including all predictors (autism and gender), covariates (age, education, income, religious attendance), and potential mediating variables (mentalizing, systemizing, Agreeableness, and Conscientiousness), mentalizing emerged as a specific, independent, and robust predictor of belief in a personal God, OR = 1.41, *Wald* = 6.01, *p* = .01 (Table 1). Older age and religious attendance also independently predicted belief in God; Agreeableness and systemizing were statistically marginal predictors (Table 1).

**Table 1:** Logistic Regression Model Predicting High Belief in God (Study 3, N = 706).

Variable	*odds ratio*	95% CI	*Wald*	*P*
Mentalizing (Empathy Quotient)	1.41	1.07–1.86	6.01	.01
Systemizing	.83	.66–1.03	2.93	.09
Agreeableness	1.26	.98–1.61	3.23	.07
Conscientiousness	1.14	.91–1.43	1.30	.25
Autism Spectrum (mediated)	1.09	.85–1.39	.51	.42
Gender (female vs. male) (mediated)	.78	.50–1.22	1.14	.29
Education	.94	.76–1.16	.36	.55
Income	.88	.71–1.10	1.17	.28
Age	1.38	1.11–1.70	8.66	.003
Rel. Attendance	5.53	4.24–7.20	160.36	.0001

### Study 4

Study 4 again replicated our findings in another national, broad sample of American adults, and controlled for four different covariates (age, education, frequency of religious attendance, and a new 3-item measure of interest in math, science, and engineering, or IMSE). In addition to the Empathy Quotient, we added a second distinct measure of mentalizing-the performance-based Reading the Mind in the Eyes or Mindreading task [32].

**Figure 3:**
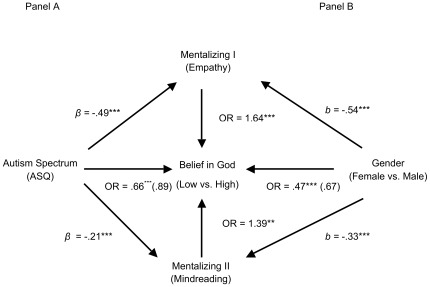
Study 4: Two distinct measures of mentalizing mediated the effects of both autism spectrum (A) and gender (B) on belief in God (*N* = 452). *p<.05, **p<.01, ***p<.001. Note. OR = odds ratio; *β* = standardized beta; b = unstandardized beta. Values in parentheses are mediated effects. Autism Analysis Covariates: Gender, Age, Education, Religious attendance, Interest in math, science, engineering. Gender Analysis Covariates: Autism Spectrum, Age, Education, Religious attendance, Interest in math, science, engineering.

In a logistic regression model, the autistic spectrum inversely predicted belief in God, controlling for gender and the four covariates: for each standard deviation increase in autism scores, participants were only 66% as likely to strongly endorse God, OR = .66, 95% CI = .53, .84, *Wald* = 12.11, *p* = .001. The two distinct mentalizing measures significantly and independently mediated this relationship: b_EmpathyIndirect_ = −.25, 95% CI = −.41, −.10, and b_MindreadingIndirect_ = −.07, 95% CI = −.14, −.02 (Fig. 3A).

A gender gap was again observed: men were 47% as likely as women to strongly endorse God, OR = .47, 95% CI = .30, .75, *Wald* = 10.07, *p* = .002, controlling for all four covariates and the autism spectrum. As hypothesized, both mentalizing measures independently mediated this gender effect: b_EmpathyIndirect_ = .27, 95% CI = .12, .45, and b_MindreadingIndirect_ = .11, 95% CI = .02, .25 (Fig. 3B). In a comprehensive logistic regression model, the two mentalizing measures emerged as significant predictors. Lower education and religious attendance also independently predicted belief in God (Table 2).

**Table 2:** Logistic Regression Model Predicting High Belief in God (Study 4, N = 452).

Variable	*odds ratio*	95% CI	*Wald*	*P*
Mentalizing I (Empathy Quotient)	1.64	1.24–2.19	11.79	.001
Mentalizing II (Mindreading)	1.39	1.09–1.79	6.92	.009
Autism Spectrum (mediated)	.89	.68–1.17	.68	.41
Gender (female vs. male) (mediated)	.67	.40–1.09	2.62	.11
Education	.80	.63–1.01	3.52	.06
Age	1.13	.90–1.42	1.15	.28
Rel. Attendance	3.68	2.82–4.79	92.63	.0001
Interest in Math, Science, Engineering	1.02	.80–1.31	0.03	.85

### General Discussion

We found new evidence for an inverse link between the autism spectrum and belief in God that was explained by mentalizing, as predicted by cognitive theories of religion [10]–[13]. Mentalizing also explained the widely observed [24] gender gap in religious belief. Our findings should be interpreted with some caution; although the results held controlling for several key socio-demographic characteristics, further research conducted in other cultural contexts should assess generalizability of findings [33]. Most of the measures were self-report (or observer-report in Study 1), which are known to have their limitations. Moreover, the correlational natures of the observations are another limitation and preclude definite causal inferences without further experimental research. Nevertheless, results were robust to various methodological checks, including different sampling strategies, alternative measures of autism, mentalizing, and religious belief, and the inclusion of several theoretically relevant control variables addressing several alternative accounts.

Specifically, one alternative is that high levels of autism cause adjustment difficulties in social situations, leading to lower levels of religious attendance, which in turn reduce religious belief. Contrary to this prediction, the effect of autism on belief in God remained significant after controlling for religious attendance (Studies 3–4), and disappeared only after controlling for mentalizing. This demonstrates that the effect of autism on belief exists even after removing the considerable overlap between belief in God and religious attendance. Relatedly, the relationship between the autism spectrum and belief cannot be solely a by-product of the more challenging social circumstances of autistic individuals, as identical patterns emerged when autism was measured as a continuous variable in a non-clinical sample of university students sharing similar social circumstances (Study 2).

A second possible alternative is reverse-causation: that religious involvement somehow causes higher levels of mentalizing, which in turn predict low scores on the autism spectrum. One causal path for this alternative is that belief in God encourages greater social involvement in religious groups and activities, which in turn increases mentalizing tendencies and decreases the likelihood of being on the autism spectrum. This interpretation did not receive support in two studies, because holding constant frequency of religious attendance did not eliminate the effect of mentalizing on belief in God. Moreover, it fails to account for the gender findings (belief in God cannot cause gender), whereas the mentalizing hypothesis parsimoniously explains both the autism and gender effects. Another reverse-causation pathway is that religious involvement leads to greater levels of mentally-simulated social engagement with supernatural agents believed to have elaborate mental states, which in turn encourages more mentalizing, and lower autism scores. Future research could test this hypothesis, but we note that this alternative pathway is compatible with the hypothesis that mentalizing deficits constrain religious belief.

Third, it is possible that the autism spectrum is associated with interest in math, science, and engineering (IMSE), which in turn reduces religious belief. However, Study 4 statistically controlled for IMSE, which did not independently predict belief in God (Table 2). Similarly, systemizing, a variable closely linked to IMSE, failed as a mediator (Studies 2–3). Fourth, the link between autism and low belief in God was not explained by general intelligence: autism remained a significant predictor of low belief in God even after statistically controlling for IQ (Study 1), and education (Studies 3–4), which is typically correlated with IQ. Fifth, the two basic personality dimensions that are most reliably predictive of religiosity, Agreeableness and Conscientiousness [30], similarly failed as mediators.

Cognitive approaches to religion emphasize that a reliably developing social cognitive mechanism-mentalizing or theory of mind-is a key foundation that supports the intuitive understanding of God or gods. Present findings bolster this hypothesis, and further demonstrate that mentalizing deficits undermine not only intuitive understanding of God, but belief as well. Furthermore, these findings suggest one reason why, despite rich sociocultural diversity, key aspects of religion reoccur across history and cultures. Additionally, the robust gender gap in religious belief has been recognized for decades, although its origins continue to be vigorously debated [24]–[26]. Our findings contribute to this debate by providing an important and previously overlooked psychological explanation for the overrepresentation of men among disbelievers.

Finally, we emphasize that our data do not suggest that religious disbelief solely arises through mentalizing deficits; multiple psychological and socio-cultural pathways likely lead to a complex and over-determined phenomenon such as disbelief in God or gods. Therefore, mentalizing deficits are one pathway among several to disbelief. Analytic cognitive processing that suppresses or overrides the intuitions that make theism cognitively compelling [34] and exposure to secular cultural contexts lacking cues that one should believe in God or gods [35] also likely promote religious disbelief. In other words, the present results suggest that disbelief can result from mentalizing deficits, but it can also arise from multiple other sources, holding constant mentalizing tendencies.

A complete scientific account of religious belief and disbelief therefore requires consideration of not only cognitive underpinnings such as mentalizing and other core cognitive biases such as dualistic intuitions and teleological or purpose-driven thinking [12], [36]. Equally important in explaining their cultural prevalence, supernatural agent beliefs­once cognitively available-can be co-opted for motivational and social functions, because of both their palliative effects on existential anxieties [1] and their facilitative effects on cooperation in large, anonymous groups in a cultural evolutionary process [37], [38]. Finally, the prevalence and content of supernatural agent beliefs, although constrained by core social cognitive capacities, respond to and fluctuate with socio-demographic conditions across time and cultures [39]. Within this broader theoretical landscape, these studies present new evidence for a social cognitive mechanism underlying one source of individual differences in religious belief.

## Materials and Methods

### Study 1

#### Participants

We recruited 12 autistic and 13 neuro-typical adolescents from the same South Florida neighbourhood. Participants were matched on key demographic and socio-economic variables (see Table 3). One additional autistic participant was excluded from analyses for failure to answer religious belief items. Families of autism-diagnosed children were contacted through local autism organizations. Autistic participants were diagnosed by registered clinicians based on DSM-IV criteria and were free of additional diagnoses.

**Table 3:** Demographic and socio-economic background information in Study 1.

Group	Age	Gender	Race/ethnicity	Parents' Religious Affiliation	Parents' Education
Neuro-typical (*n* = 13)	*M* = 12.6	12 Male	11 Caucasian	7 Protestant	*M* = 4.80
		1 Female	1 Asian	3 Catholic	
			1 Hispanic	1 Catholic/Other	
				2 Jewish	
Autistic (*n* = 12)	*M* = 13.7	11 Male	11 Caucasian	2 Protestant	*M* = 4.90
		1 Female	1 Hispanic	4 Catholic	
				3 Jewish	
				1 Jewish/Other	
				1 Other Religion	
				1 No Religion	

*Note. Parents' education* was defined as the average educational attainment of both parents (range 2–6; 2 = some college, 3 = college certificate, 4 = some university, 5 = university degree, 6 = graduate degree). *Parents' Religious Affiliation* refers to both parents' stated religion.

#### Dependent Measure: Belief in God

Participants rated their agreement (1–7) with four different statements (I believe in God; When I am in trouble, I find myself wanting to ask God for help. Reversed-coded items: When people pray they are only talking to themselves; I don't really spend much time thinking about my religious beliefs). One additional item (“I just don't understand religion”) was dropped, because it correlated poorly with the overall scale in this sample, leaving a four-item Intuitive Belief in God scale (*α* = .65, *M* = 5.03, *SD* = 1.37). (Retaining this item did not significantly alter the overall pattern of results). In previous research [34], this measure correlated very highly with other scales measuring religious devotion, such as the Intrinsic Religiosity Scale [40] (*r* = .65, p<.001), and the Spiritual Well-Being Scale [41] (*r* = .82, p<.001). Belief in God was non-normally distributed and negatively skewed (Kolmogorov-Smirnov *p* = .08, skewness = −.82). Therefore, this variable was median-dichotomized into high believers (60%) and low believers (40%).

#### Predictor Measures: Autism, Mentalizing, and IQ

Parents rated their adolescent child using the parental version of the 50-item Autism Spectrum Quotient [23] (ASQ) (*α* = .91, *M* = 20.67, *SD* = 11.67). Representative items from the parental version of the ASQ: “S/he is fascinated by numbers,” “S/he is good at social chit-chat” (reverse-scored). The autistic group (*M* = 30.82, *SD* = 7.39) scored significantly higher on parental ratings of autism than did the neuro-typical control group (*M* = 12.08, *SD* = 6.42), *t*(22) = 6.65, *p* = .0001, *Cohen's d* = 2.84, validating the clinical diagnoses. Mentalizing was assessed by the parents' ratings of their child, using the short, 22-item version of the Empathy Quotient [28] (*α* = .97, *M* = 17.50, *SD* = 12.75), which measures perspective taking, interest in inferring others' beliefs and desires, and understanding emotion. Representative items from the parental version of the Empathy Quotient: “My child often finds it difficult to judge if someone is rude or polite” (reverse scored), “My child is good at understanding how others feel and what they are thinking.” We assessed General Intelligence (IQ) with the age-appropriate Kauffman Brief Intelligence Test, 2^nd^ Edition [42], which consists of both verbal and non-verbal subtests (*M* = 92.31, *SD* = 21.05). None of the items in any of the predictor measures had any religious content. Autism scores were not reliably associated with IQ, *r* (25) = −.275, *p* = .19.

### Study 2

#### Participants

In exchange for extra credit in psychology courses, 327 Canadian students (*M_Age_* = 20.0, 66% female) completed a web-based questionnaire. For information about participants' religious backgrounds, see Table 4.

**Table 4:** Demographic and socio-economic background information in Studies 2–4.

Study 2	*Religious affiliations*: Catholic and Protestant Christians (37.1%), Buddhists (5.4%), Muslims (2.9%), Atheists (13.7%), Agnostics (11.7%), participants who listed “None” (26.0%), Hindus (.9%), Sikhs (2.0%), and one Jew (.3%).
(*N* = 327)	
Study 3	*Religious affiliations*: Catholic and Protestant Christian (48.5%), Jewish (2.5%), Buddhists (1.3%), Hindu (2%), Muslim (.7%), Atheists (11.6%), Agnostic (14.2%), “None” (14%), and a variety of “Other” faiths (5.2%).
(*N* = 706)	*Educational backgrounds*: .8% less than high school, 10.8% high school or equivalent, 37.7% some university or college, 38.4% completed university or college, 12.3% completed a postgraduate degree).
	*Ethnic/racial background*: White/Caucasian (76%), African American (5.8%), Hispanic (5.7%), East Asian (4.6%), other (7.9%).
	*Reported annual income*: range: <$10 K–>$100 K, with the mean in the $30 K–$40 K range.
	*Religious attendance*: (range: never – more often than weekly), with the median falling between once/twice per year and every other month.
Study 4	*Religious affiliations*: Christians (70%), Jews (4%), Buddhists (1%), Muslims (1%), Atheists (4%), Agnostics (5%), “Nones” (10%), and a variety of “Other” faiths (5%).
(*N* = 452)	*Educational backgrounds*: (3% less than high school, 20% high school or equivalent, 36% some university or college, 27% completed university or college, 6% some postgraduate education, and 8% completed a postgraduate degree).
	*Religious attendance*: “I attend church (or other religious services) often” (Range: 1–7, *M* = 3.50, *SD* = 2.42).

#### Dependent Measure: Belief in God

We used the same 5-item belief in God measure used in Study 1 (*α* = .81, *M* = 27.70, *SD* = 8.06). Participants rated their agreement (1–7) with five different statements. Because the Kolmogorov-Smirnov test is oversensitive in large samples, we assessed normality with visual inspection and other tests of normality (skewness = .08), which indicated that this measure was approximately normally distributed in this sample. *Predictor Measures: Autism Spectrum and Gender*. In addition to gender, we measured the widely used Autism Spectrum Quotient (*α* = .73, *M* = 16.83, *SD* = 5.96) a 50-item questionnaire that detects normal individual differences in autism for people having normal levels of intelligence [23]. Sample items: “I prefer to do things the same way over and over again.” “I am fascinated by numbers.” “I find social situations easy” (reverse scored).

#### Potential Mediators: Mentalizing and Systemizing

We measured mentalizing with the short self-report version of the Empathy Quotient [28] (22 items, *α* = .88, *M* = 23.06, *SD* = 7.75), which assesses the ability to identify the mental states of others and to react appropriately to them (sample items: “I often find it difficult to judge if someone is rude or polite” (reverse scored). “I am good at predicting how someone will feel.”). We measured systemizing with the short version of the Systemizing Quotient [28] (25 items, *α* = .84, *M* = 16.37, *SD* = 7.99). This scale measures aptitude for, and interest in, reasoning about mechanical and physical objects and processes. Sample items: “I am fascinated by how machines work.” “I find it difficult to understand information the bank sends me on different investment and saving systems” (reverse scored).

### Study 3

#### Participants

A broad national sample of Americans was drawn from a US-based online survey tool (Mechanical Turk, Amazon.com). A total of 725 participants were recruited, however incomplete data reduced the sample to *N* = 706, Age: 18–88, *M* = 30.23, *SD* = 10.65, 63% female. Participants came from all 50 states, and had diverse religious backgrounds, educational attainment, levels of income, and levels of religious attendance (see Table 4 for further demographic information).

#### Dependent Measure: Belief in a personal God

Participants rated their agreement (0–6) with 10 items selected from the previously validated and widely-used Spiritual Well Being Scale [41]. These items measure belief in, and a perceived relationship with, a personal God (*α* = .94, M = 43.88, SD = 18.55). Representative items: “I have a personally meaningful relationship with God,” “I believe that God is concerned about my problems.” Reverse Scored: “I believe that God is impersonal and not interested in my daily situations.” “I don't find much satisfaction in private prayer with God.” Visual inspection revealed a bimodal distribution (Kurtosis = −1.36). Therefore, the measure was median-dichotomized into low believers (51.6%) and high believes (48.4%).

#### Predictor Measures: Autistic Spectrum and Gender

In addition to identifying their gender, participants completed the same 50-item self-report version of the ASQ (*α* = .80, *M* = 19.10, *SD* = 7.03).

#### Potential Mediators: Mentalizing, Systemizing, Agreeableness, Conscientiousness

We measured mentalizing and systemizing with the same measures used in Study 2 (Empathy: *α* = .91, *M* = 22.87, *SD* = 9.46; systemizing: *α* = .87, M = 21.53, SD = 9.60). Agreeableness and Conscientiousness were measured using the relevant facets of a standard self-report instrument measuring basic personality dimensions [29].

#### Control Measures

We measured age, educational attainment, income level, and frequency of religious attendance. Older age, lower educational attainment, and lower income are often found to be associated with religious belief. The frequency of religious attendance measure was included to subject our hypotheses to a more stringent test, by disentangling belief in God from an associated inclination towards religious social participation.

### Study 4

#### Participants

A broad national sample of 452 Americans (Age: 18–84, M = 43.06; 50.7% female) was drawn from a paid subject pool administered by a US-based survey company (www.Zoomerang.com). Although this was not a representative sample, participants' ethnic and religious backgrounds were roughly similar to the general American population, and there was a wide range of educational attainment and religious attendance (see Table 4 for further socio-demographic background information).

#### Dependent Measure: Belief in God

The same 5-item scale was used from Studies 1–2 (*α* = .82, *M* = 25.71, *SD* = 7.46). This American sample yielded a markedly non-normal distribution of belief in God scores, as levels of belief in God were generally high (skewness = −.56, kurtosis = −.51). This variable was therefore median-dichotomized into high believers (51% of sample) and low believers (49% of sample).

#### Predictor Measures: Autistic Spectrum and Gender

In addition to identifying their gender, participants completed the same 50-item self-report version of the ASQ [23] (*α* = .71, *M* = 18.60, *SD* = 6.04).

#### Potential Mediators: Mentalizing

First, participants again completed the self-report version of the Empathy Quotient from Studies 2–3 (*α* = .90, *M* = 22.21, *SD* = 8.85). Second, participants completed the revised Reading the Mind in the Eyes (RME) task [32] (*α* = .71, *M* = 24.43, *SD* = 4.91), which consists of a series of 36 pictures of peoples' eyes. Participants are instructed to select which of four words best describes what the person in the picture is thinking or feeling. This task has been used to detect individual differences in advanced adult mentalizing [32]. This latter measure shares neither conceptual resemblance nor method variance with the belief in God measure. The two mentalizing measures were related, but by no means redundant, *r* (450) = .25, *p*<.001, and served as independent potential mediators in a multiple mediation model.

#### Control Measures

We again measured and controlled for age, educational attainment, frequency of religious attendance, and added a 3-item measure of interest in math, science, and engineering (IMSE, α = .69, on a 1–7 scale). IMSE was included to assess the possibility that the relationship between autism and belief in God, or gender and belief in God, are byproducts of greater levels of scientific interest among those high on the autism spectrum.
